# Editorial: Prevention and treatment advancements in diabetic retinopathy

**DOI:** 10.3389/fendo.2026.1807742

**Published:** 2026-02-18

**Authors:** Tomislav Bulum, Tomislav Jukić, Domagoj Ivastinović, Mayank Choubey, Miroslav Stamenković, Martina Tomić

**Affiliations:** 1 Department of Diabetes and Endocrinology, Vuk Vrhovac University Clinic for Diabetes, Endocrinology and Metabolic Diseases, Merkur University Hospital, Zagreb, Croatia; 2 School of Medicine, University of Zagreb, Zagreb, Croatia; 3 Department of Ophthalmology, University Hospital Centre Zagreb, Zagreb, Croatia; 4 Department of Ophthalmology, Medical University Graz, Graz, Austria; 5 Department of Foundations of Medicine, NYU Grossman Long Island School of Medicine, Mineola, NY, United States; 6 University Medical Center Zvezdara, Eye Clinic, Belgrade, Serbia; 7 Department of Diabetic Eye Complications, Vuk Vrhovac University Clinic for Diabetes, Endocrinology and Metabolic Diseases, Merkur University Hospital, Zagreb, Croatia

**Keywords:** biomarkers, diabetic macular edema, diabetic retinopathy, prevention, type 2 diabetes, vascular endothelial growth factor

Diabetes is a global pandemic with almost 600 million adults living with diabetes in 2024 (1, 2). High blood glucose levels have harmful effects on small vessels and neuroretinal structures, leading to the development of diabetic retinopathy (DR), a microvascular complication of diabetes, which is currently a leading cause of preventable blindness in working-age adults (3). While the majority of patients with diabetes will develop DR, it is important to stress that about 10% of them will develop vision-threatening forms of DR, such as proliferative DR (PDR) and diabetic macular edema (DME) (4). While there is a trend of decreasing the incidence of the most severe forms of DR in developed countries, the risk of vision-threatening forms of DR is still high, and the overall prevalence of DR is increasing due to the global diabetes epidemic (5). In light of these considerations, further action is required to minimize the burden of vision loss and disability among individuals with diabetes. This Research Topic, therefore, highlights current developments and recent progress in the understanding and management of DR and DME, bringing together 18 original research articles, 5 narrative reviews, and 3 systematic reviews.

Chen et al. investigated the association between serum selenium levels and DR and collected data from 645 adults through the National Health and Nutrition Examination Survey (NHANES) between 2011 and 2016. While there was no statistically significant linear association between serum selenium levels and the probability of developing DR, a U-shaped relationship was observed. The incidence of DR is elevated in individuals with serum selenium levels that are either higher or lower than the optimal range, suggesting that selenium supplementation should be balanced to avoid excessive intake.

In addition to selenium, vitamin D has been reported to be involved in DR pathogenesis, but results have been inconsistent. He et al. explore the relationship between vitamin D level and the risk of DR in 535 adult patients with type 2 diabetes mellitus (T2DM). The results suggest that males, but not females, with DR had significantly lower vitamin D levels and a higher proportion of severe vitamin D deficiency than those without DR. In addition, total testosterone levels were significantly lower in males with DR. The findings indicate a sex-specific association between serum vitamin D levels and the risk of DR in T2DM. A significant inverse relationship was observed exclusively in men, potentially driven by a substantial decline in total testosterone levels.

Since emerging evidence suggests a potential association between depression and DR, Li et al. investigate the relationship between depression and DR using nationally representative data from the National Health and Nutrition Examination Survey (NHANES, 2011–2020). Depression was assessed using the PHQ-9, and among 1,653 participants, the weighted DR prevalence was 18.91%. Depression was independently associated with higher DR risk. These findings underscore the importance of an integrated approach that addresses both mental health and metabolic regulation in individuals with diabetes. Targeted management of depressive symptoms may help mitigate the burden of DR and enhance overall quality of life.

The most common microvascular complications in diabetes are DR and diabetic kidney disease, although the occurrence of those two diseases is not parallel. Therefore, Yin et al. tried to identify the risk factors for combining DR and diabetic kidney disease in T2DM and construct a nomogram predictive model to identify high-risk patients with DR combined with diabetic kidney disease. The study included 683 T2DM patients and identified eight independent risk factors: fibrinogen, albumin, atherogenic index of plasma, low-density lipoprotein cholesterol, body mass index, classification of DR, gender, and history of hypertension. These variables were incorporated into the development of a nomogram-based predictive model. The model may support clinical decision-making by emphasizing blood pressure control, lipid management, weight reduction, and nutritional optimization, thereby helping prevent or delay the progression of diabetic kidney disease in T2DM with DR.

Aiming further to explore the association between DR and kidney disease, Yuan et al. examined whether renal function influences surgical outcomes following pars plana vitrectomy in patients with PDR. A retrospective analysis included 128 eyes with at least 2 years of follow-up and compared patients with impaired versus normal renal function. No significant differences were found between groups regarding intraoperative procedures, postoperative complications, or reoperation rates. Although patients with impaired renal function had worse baseline visual acuity, they experienced greater long-term postoperative visual improvement. Importantly, final visual outcomes were comparable across groups, suggesting that renal insufficiency does not compromise the safety or effectiveness of vitrectomy in patients with PDR.

Dongling et al. developed and validated a universal nomogram to predict referable DR in T2DM using routinely available clinical indicators. A cross-sectional analysis of 1,830 community-based patients and external validation in 123 outpatients identified seven key predictors: serum creatinine, urea nitrogen, urine glucose, HbA1c, urinary microalbumin, diabetes duration, and systolic blood pressure. The nomogram demonstrated moderate, consistent predictive performance across the training, testing, and external validation cohorts. Decision curve analysis supported its clinical usefulness over a wide range of risk thresholds. Overall, the model offers a practical, scalable tool for early referable DR risk stratification in both community and ophthalmic settings.

Liu et al. developed a DR prediction model using machine learning and the novel Triglyceride-glucose index (TyG). Data from 3,750 patients were analyzed, and SHapley Additive exPlanations (SHAP) analysis identified TyG, insulin therapy, HbA1c, diabetes duration, and HDL as key predictors, with HDL protective. This model highlights TyG as a cost-effective, practical alternative to traditional insulin resistance measures, such as HOMA-IR, enabling accessible DR risk stratification in primary care.

In addition, a systematic review and meta-analysis from Amirashov et al. examined the association between the TyG and DR. Sixteen observational studies involving over 33,000 participants were included. Higher TyG levels were associated with increased DR risk, particularly when analyzed as a continuous variable. However, substantial heterogeneity and potential publication bias were observed, especially in categorical analyses. Subgroup and meta-regression analyses identified male proportion as a key source of heterogeneity. These findings suggest a possible link between TyG and DR, though inconsistent thresholds and bias limit immediate clinical application.

She et al. investigated the association between the albumin-to-neutrophil-lymphocyte ratio (ANLR), a marker of nutritional and inflammatory status, and DR using NHANES data (6,279 participants) and an external validation cohort (212 participants). Higher ANLR levels were associated with significantly lower DR risk, with a nonlinear L-shaped relationship: rapid risk reduction below an ANLR of 20 and a plateau thereafter. External validation confirmed these findings and demonstrated a decrease in ANLR across DR severity stages. Subgroup analyses indicated more substantial protective effects in females, individuals with higher BMI, and those with favorable lipid profiles. ANLR may serve as an accessible biomarker for DR risk stratification and early intervention.

Xi et al. investigated the association between sleep patterns and DR in T2DM and the mediating role of metabolic factors. Among 2,433 participants, longer nighttime sleep and extended daytime napping were both linked to a higher risk of DR, with combined long sleep and naps showing the strongest association. These relationships varied by sex and diabetes duration, with male patients and those with shorter disease duration being particularly affected. Mediation analyses indicated that metabolic factors, including blood pressure, BMI, and HbA1c, partially explained these associations, suggesting a combined behavioral and metabolic pathway in DR development.

Insulin resistance is a central condition associated with the development of T2DM and is also a risk for the development of chronic diabetic complications. Gao et al. used estimated glucose disposal rate (eGDR) as a surrogate marker of insulin resistance and investigated the association between the eGDR and DR in 2,327 patients with T2DM, including its potential value as a predictive marker. After controlling for confounding factors, the risk of DR decreased by 40.4% for everyone standard deviation increase in eGDR. The results of the study suggest that eGDR might serve as a target for DR prediction and intervention.

Lucio et al. estimated the economic burden of avoidable blindness caused by DME in Ecuador. Using a one-year retrospective cost-of-illness approach, it compared the costs of treating established blindness with those of preventing vision loss through timely intervention. In 2023, blindness-related costs reached USD 259.7 million, primarily driven by productivity losses, whereas prevention costs were USD 108.5 million annually. Treating blindness was substantially more expensive than prevention, highlighting the economic and societal benefits of investing in early detection and treatment policies for diabetes-related visual impairment.

A retrospective study from Han et al. compared 25G 10,000 cpm beveled-tip microincision vitrectomy (MIVS) with conventional 25G flat-tip MIVS in 58 eyes with PDR requiring epiretinal membrane removal. The beveled-tip group achieved more efficient membrane cutting, required fewer instrument exchanges, and had shorter total and vitrectomy surgery times, with fewer intraoperative hemostasis maneuvers. Postoperative outcomes, including visual acuity and intraocular pressure, were similar between groups. These findings suggest that 25G 10K beveled-tip MIVS improves surgical efficiency while maintaining safety and effectiveness in PDR management.

Li et al. examined the relationship between red blood cell count and DR in 413 patients with T2DM in an exploratory study. Using training and validation cohorts, a nomogram was developed incorporating red blood cell, serum creatinine, diabetes duration, diabetic peripheral neuropathy, and diabetic kidney disease. The model showed good predictive performance (AUCs of 0.765 in training and 0.707 in validation) and calibration, with decision curve analysis supporting clinical utility. Results suggest an inverse association between red blood cell and DR risk, providing a preliminary framework for DR risk assessment pending further prospective validation.

Garcia et al. evaluated the impact of the COVID-19 pandemic on the Andalusian Program for Early Detection of Diabetic Retinopathy (APDR) from 2018 to 2023. During 2020–2021, both new patient registrations and retinal imaging decreased sharply, but activity rebounded in 2022, surpassing pre-pandemic levels. Despite these disruptions, the proportion of vision-threatening DR cases remained stable throughout the pandemic and post-pandemic period. The findings highlight the program’s resilience and effective recovery, though continued long-term monitoring is needed to assess potential impacts on late-stage complications.

Cui et al. developed and validated a nomogram to predict individual responses to combined anti-VEGF and laser therapy in patients with severe non-PDR. A retrospective cohort of 280 patients was analyzed, with a 12-month composite response as the primary outcome. Four predictors—glycated hemoglobin variability, fluorescein angiography non-perfusion area, baseline DR severity, and serum albumin—were retained. The nomogram demonstrated intense discrimination (AUC of 0.821 in derivation and 0.754 in validation), good calibration, and clinical utility. This tool enables personalized risk stratification, guiding treatment intensification for patients at higher risk of suboptimal response and supporting precision management in DR.

In addition, Wang et al. analyzed the relationship between OCT biomarkers and outcomes in 113 patients with DME treated with anti-VEGF therapy. Patients were classified as cystoid macular edema (CME), diffuse retinal thickening (DRT), or serous retinal detachment (SRD). CME patients had the worst baseline visual acuity, while DRT showed the fewest hyperreflective foci (HRF). Anti-VEGF therapy reduced HRF across all subtypes, with baseline inner and outer retinal HRF and central macular thickness serving as independent predictors of visual prognosis. Findings suggest that OCT biomarkers can guide subtype-specific treatment and monitoring, improving long-term visual outcomes in DME.

Li et al. investigated molecular drivers of DR and the therapeutic potential of the reactive sulfur donor PSCP. Transcriptomic analyses in diabetic mice identified mitochondrial antioxidant enzymes IDH2 and MGST1 as consistently dysregulated. In ARPE-19 cells, oxidative and carbonyl stress impaired mitochondrial function and suppressed these enzymes. PSCP treatment activated the Keap1-Nrf2 pathway, restoring IDH2 and MGST1 expression, preserving mitochondrial integrity, and improving cell survival. These findings highlight IDH2 and MGST1 as key stress-responsive targets and suggest that PSCP may be a potential protective therapy in DR.

Feng et al. explored the association between free triiodothyronine (fT3) levels and DR in 3,703 patients with T2DM. Longitudinal follow-up of 1,476 patients revealed that moderate fT3 levels reduced the risk of DR onset or progression, especially in individuals with poor glycemic control, longer diabetes duration, or older age. Mendelian randomization suggested a potential protective effect of higher fT3 on DR and highlights fT3 as a possible biomarker for DR risk stratification and prevention.

A systematic review and meta-analysis from Jiang et al. compared the efficacy of subthreshold micropulse laser (SML) combined with anti-VEGF therapy versus anti-VEGF monotherapy in patients with DME. Thirteen studies involving 805 eyes were included. While ETDRS visual acuity did not differ significantly between groups, LogMAR visual acuity showed greater improvement with combination therapy at 6 and 12 months. Patients with baseline central macular thickness (CMT) below 400 µm experienced greater CMT reduction with combined treatment, whereas no difference was observed in those with CMT above 400 µm. Importantly, combination therapy significantly reduced the annual number of anti-VEGF injections, potentially lowering treatment burden and costs.

Oxidative stress plays a central role in the pathogenesis of DR. He et al. explained in a review article how excess reactive oxygen species damage retinal vessels and neurons through metabolic pathways such as PKC activation, AGE formation, and mitochondrial dysfunction, leading to inflammation and apoptosis. Epigenetic changes further amplify these effects. Therapeutic strategies targeting oxidative stress, including antioxidants, natural compounds, and activation of Nrf2 and SIRT1 pathways, show promise in preclinical studies, although further clinical validation is required.

Wang et al. describe new potential biomarkers in DR. Early detection of DR is hindered by subtle symptoms and limited screening resources. Omics technologies—genomics, transcriptomics, proteomics, and metabolomics—are increasingly used to identify biomarkers and clarify disease mechanisms. Recent advances in omics research have improved understanding of DR pathophysiology, supported earlier diagnosis, and promoted personalized treatment strategies, contributing to better prevention and reduced global impact of the disease.

Current treatments of DR, such as anti-VEGF therapy, are invasive and less effective in the early stages, underscoring the urgent need for novel strategies targeting early intervention. A review article by Chen et al. highlights microRNAs (miRNAs) as central regulators linking hyperglycemia to oxidative stress, inflammation, neurodegeneration, and vascular dysfunction in DR. Dysregulated miRNAs exhibit stage-specific patterns in biofluids, making them promising non-invasive biomarkers. Moreover, miRNA-based therapies, supported by advanced profiling technologies, offer potential for early intervention, precision management, and prevention of DR progression.

A comprehensive review from Zong et al. examines the evolving landscape of monoclonal antibody (mAb) therapy in DR management, highlighting anti-VEGF agents and emerging strategies targeting the angiopoietin–Tie2 pathway. Innovations such as high-dose aflibercept, port delivery systems, and bispecific antibodies like faricimab have expanded treatment options. Although anti-inflammatory antibodies remain experimental, monoclonal antibody therapy has transformed DR management, while ongoing challenges such as treatment burden and cost underscore the need for personalized and innovative approaches.

A systematic review by Huang et al. evaluated published DR prediction models to identify the most useful for clinical practice. Fifteen studies were included, with model performance ranging from 0.70 to 0.96. Common predictors included diabetes duration, age, HbA1c, serum creatinine, and urinary albumin–creatinine ratio. Although several models showed good applicability, all were deemed to have a high risk of bias. The findings highlight the need for more transparent model development, larger datasets, and robust multicenter external validation to improve reliability and clinical utility.

Glucagon-like peptide-1 (GLP-1) receptor agonists are now a fundamental class of drugs used in the treatment of T2DM because of their cardiorenoprotective properties and weight-reducing effects (6). However, their impact on DR appear to be neutral, or in some cases, even harmful (7). A systematic review and meta-analysis from Alwafi et al. aimed to clarify the association between GLP-1 receptor agonist use and the development or progression of DR. Thirty-nine studies were reviewed, with 23 included in the meta-analysis. Overall, GLP-1 receptor agonist use was not significantly associated with DR risk compared with other therapies. Subgroup analyses by study design, sample size, and risk of bias yielded consistent non-significant results, with a modest trend toward reduced risk in randomized trials.

The collective evidence presented in this Research Topic provides a comprehensive overview of recent advances in research on DR and DME in T2DM and emphasizes the need to improve our understanding and management of these vision-threatening complications.”
